# Transportation Modes Classification Using Sensors on Smartphones

**DOI:** 10.3390/s16081324

**Published:** 2016-08-19

**Authors:** Shih-Hau Fang, Hao-Hsiang Liao, Yu-Xiang Fei, Kai-Hsiang Chen, Jen-Wei Huang, Yu-Ding Lu, Yu Tsao

**Affiliations:** 1 Department of Electrical Engineering, Yuan Ze University, Taoyuan 320, Taiwan; shfang@saturn.yzu.edu.tw (S.-H.F.); s1020636@ee.yzu.edu.tw (H.-H.L.); mike50303@yahoo.com.tw (Y.-X.F.); 2 Department of Electrical Engineering, National Cheng Kung University, Tainan 701, Taiwan; evin761015@hotmail.com (K.-H.C.); jwhuang@mail.ncku.edu.tw (J.-W.H.); 3 Research Center for Information Technology Innovation, Academia Sinica, Taipei 115, Taiwan; s96387799@gmail.com

**Keywords:** transportation mode, big data, machine learning, sensor, smart phone, classification

## Abstract

This paper investigates the transportation and vehicular modes classification by using big data from smartphone sensors. The three types of sensors used in this paper include the accelerometer, magnetometer, and gyroscope. This study proposes improved features and uses three machine learning algorithms including decision trees, K-nearest neighbor, and support vector machine to classify the user’s transportation and vehicular modes. In the experiments, we discussed and compared the performance from different perspectives including the accuracy for both modes, the executive time, and the model size. Results show that the proposed features enhance the accuracy, in which the support vector machine provides the best performance in classification accuracy whereas it consumes the largest prediction time. This paper also investigates the vehicle classification mode and compares the results with that of the transportation modes.

## 1. Introduction

In recent years, smartphones are becoming more and more popular. Each phone typically contains a variety of sensors, such as a GPS (Global Positioning System) sensor, a magnetometer, and a gyroscope sensor, etc. Therefore, it is easy to get a large amount of sensor data from smartphones. This paper utilizes the information from such sensors to detect different types of transportation modes. Classifying a person’s transportation mode plays a crucial rule in performing context-aware applications. Using sensors embedded in smartphones has been recognized as a good approach.

Much literature has studied this issue. For example, Elhoushi et al. [1] proposed an algorithm for indoor motion detection such as walking, sitting, standing, etc. They used the accelerometer triad, the gyroscope triad, the magnetometer triad, and the barometer information as the input sensors. Hemminki et al. [2] proposed an algorithm to use smartphones to detect five transportation modes, including bus, train, metro, tram and car. They used kinematic motion classifiers to distinguish whether users were walking or not. Once the motorized transportation was detected, the motorized classifier could classify the current transportation activity. Sasank et al. [3] used GPS and accelerometer data as the input data. After filtering out the noise, they built an instance-based decision tree as the classifier and used a discrete hidden Markov model to make the final decision. Ben et al. [4] collected the accelerometer data. They used the magnitudes of the 250 FFT (Fast Fourier Transform) components and the statistics of the signal as features and used genetic data analysis and SVM (Support Vector Machine) to classify the data. Yu et al. [5] used the accelerometer, magnetometer, and gyroscope as the input data and derived similar features. Transportation mode classification can be divided into two categories, location-based [6,7,8] and sensor-based approaches [9,10]. The former relies on the GPS data or wireless network information [11,12,13]. Unfortunately, the location-based methods suffer from high power consumption and may not work in some environments [14,15]. Yu et al. [5] suggests that GPS and Wi-Fi consume significant power of 30 mA and 10.5 mA, respectively, which is not suitable for handheld devices. This paper belongs to the second category, sensor-based approaches, which do not rely on GPS and do not assume unlimited power and resources [16,17]. To address the practical issues, this study proposes a low-dimensional feature and evaluates the memory usage (model size), response time (processing time and signal overlap), and general accuracy. Compared with other studies of transportation mode classification, the main contribution of this paper is two-fold. First, in additional to accuracy, we further address some practical issues of resource consumption. Second, we use large-scale big sensor data (over 1000 h) with more attributes (10 modes) to evaluate the performance.

In this paper, three types of low-power-consumption sensors include the accelerometer, magnetometer, and gyroscope. This paper has extracted the features from the time series of those sensor measurements by integrating them into the time domain and frequency domain. The experimental results are shown in the two modes of classification: transportation and vehicle mode classification based on three machine learning algorithms such as decision trees (DT), k-nearest neighbor (KNN), and support vector machine (SVM). To address the practical issues, this study proposes a low-dimensional feature and evaluates the memory usage (model size), response time (processing time and signal overlap), and general accuracy. This is the first key difference as compared to other similar studies, which focus on accuracy and use hundreds of features [1,14,17]. The results show that the accuracy of the proposed feature improves the performance. In the transportation mode classification tasks, SVM shows the best performance in accuracy compared to DT and KNN. For vehicle mode classification tasks, KNN outperforms SVM and DT.

The novelty of this paper is summarized as follows: (1) we investigate both the transportation (still, walk, run, bike, vehicle) and vehicular (motorcycle, car, bus, metro, train and high speed rail) mode classification. To the authors’ best knowledge, this is the first work investigating these complex attributes. Most existing works focus on simple user behaviors such as walking, running, jumping, etc. (2) We study suitable features for both modes under limited power and resources. Most existing works focus on accuracy only using unlimited features. For example, Elhoushi et al. [1] used 334 features while Figo et al. [18] studied various time domain and frequency domain features. To our best knowledge, the most related work to this study is Reference [5], which discussed the power consumption of different sensors and summarized seven features into low-power transportation mode detection. This study selects Reference [5] as a benchmark. In fact, the aim of this study is not to propose new statistic features, which have been well investigated. Instead, we try to select and combine useful features from existing works under the power and dimension constraints for both transportation and vehicular mode classification tasks.

The rest of this paper is organized as follows. Section 2 describes the research method, including the database, feature extraction, and classifier learning. Section 3 shows the experimental results, and Section 4 summarizes the conclusion.

## 2. Research Method

### 2.1. Database

The data were provided by HTC company, and were collected since 2012 over two years, involving 224 volunteers and totally contained 8311 h of 100 GB [5]. The data used in this study was a part of the raw data in [5], roughly 20 GB, which HTC makes them public for the academic use. The pool of participants sufficiently covered different genders (60% male), builds, and ages (20 to 63 years old). The transportation state includes 10 modes, still, walk, run, bike, motorcycle, car, bus, metro, train and high speed rail (HSR). Compared to other similar studies which use small-scale data (several or dozens hours) [1,19], such big data makes the results of this paper more convincing and general.

The database for five transportation modes is indicated by Table 1. This paper classifies the vehicular modes (i.e., motorcycle, car, bus, metro, train, and HSR) as a single mode: on a vehicle. Then, these data would be separated into training and testing data for the performance evaluation.

**Figure 1:**
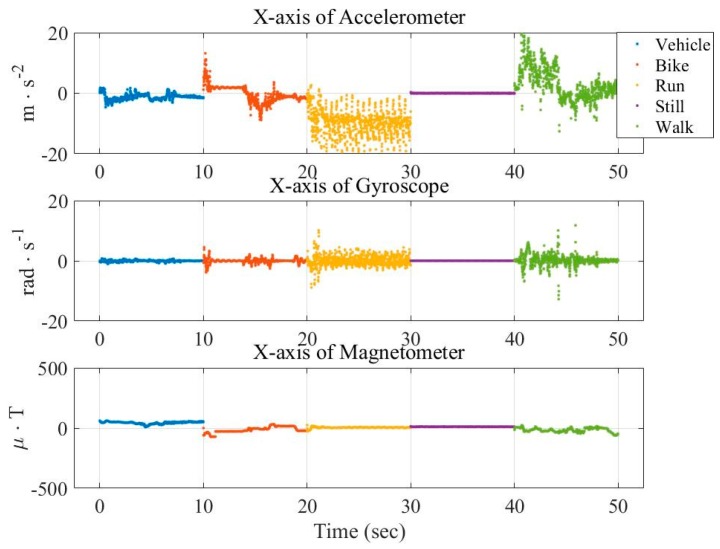
Distribution of raw data from three sensors (x-axis) in the transportation mode.

**Figure 2:**
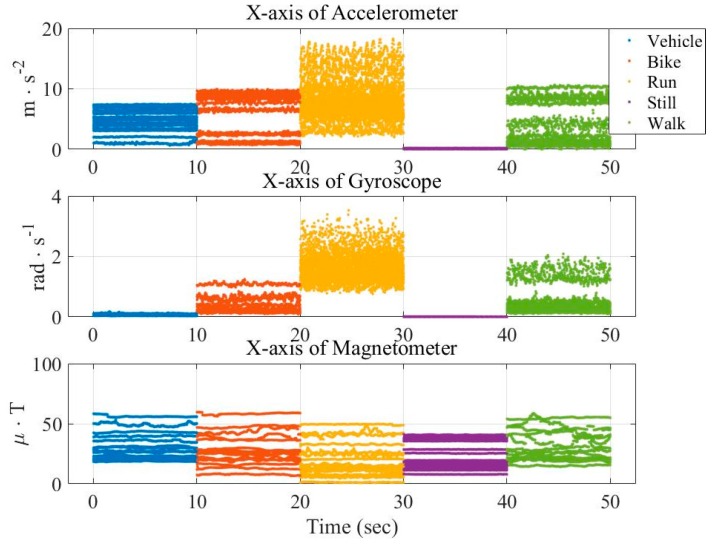
Distribution of averaged data from three sensors (x-axis) in the transportation mode.

In this paper, we attempt to visualize the big data and the corresponding features from different perspectives. Figure 1 and Figure 2 show the distribution of the raw data and averaged data, respectively, from three x-axis of sensors in the transportation mode. The raw data is randomly selected 10 s, while the averaged data is obtained by computing the absolute value of the 1000 min average from the large dataset. These figures show that the long-term statistic is different from that of the raw data, verifying the importance of the temporal processing in the features.

**Figure 3:**
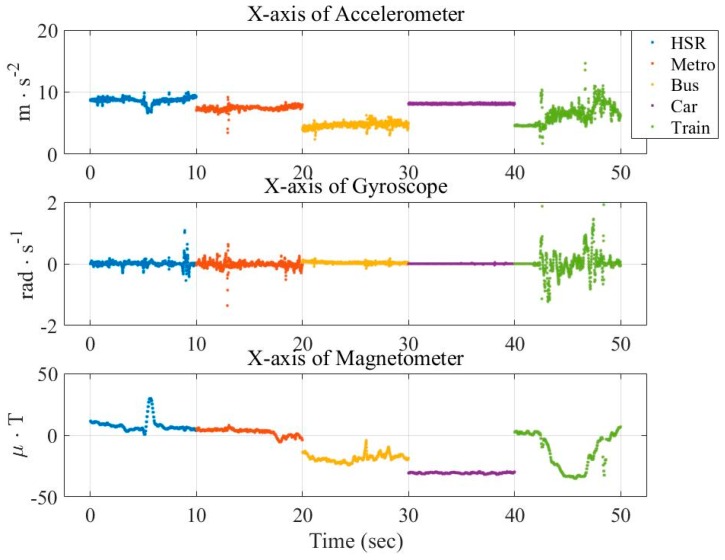
Distribution of raw data from three sensors (x-axis) in the vehicular mode.

**Figure 4:**
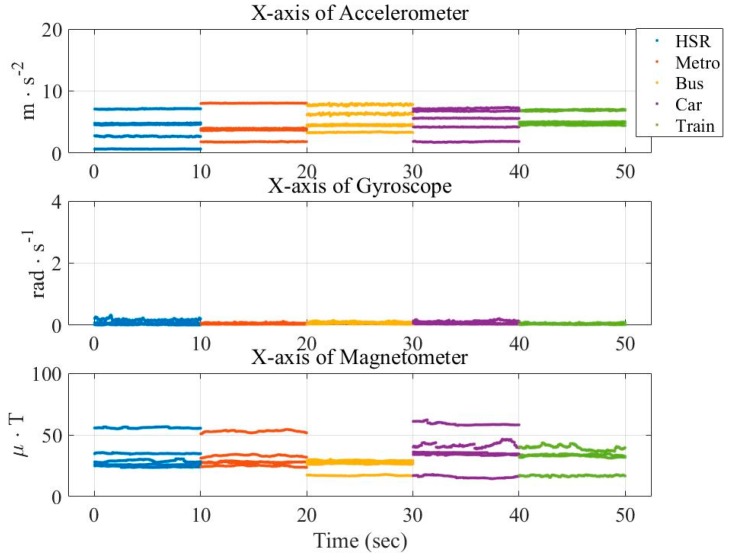
Distribution of averaged data from three sensors (x-axis) in the vehicular mode.

Similarly, Figure 3 and Figure 4 show the vehicular cases. These figures show that the measurements are not discriminate as that in Figure 1 and Figure 2. This demonstrates the difficulty of vehicle mode detection.

### 2.2. Feature Extraction

With database from Section 2.1, this paper can integrate these data into diversified features. In this paper, 512 samples are integrated into a frame, and a moving window with 75% overlap is used to generate the next frame. In this setup, the monitoring period of each frame is 17.06 s. The 75% overlap means that we reused the 17.06 × 75% = 12.8 s data as the next frame to smooth the data continuity and to reduce the system delay. Then, these frames would be transformed into various features. Because we select Reference [5] as a baseline, the seven features used in Reference [5] are listed below:

(1) Average of the accelerometer’s magnitude.
(2) Standard deviation of the accelerometer’s magnitude.
(3) The highest FFT value of the accelerometer.
(4) The ratio between the highest and the second-highest FFT value of the accelerometer.
(5) Standard deviation of the magnetometer’s magnitude.
(6) Standard deviation of the gyroscope’s value.
(7) Average of the gyroscope’s value.

**Figure 5:**
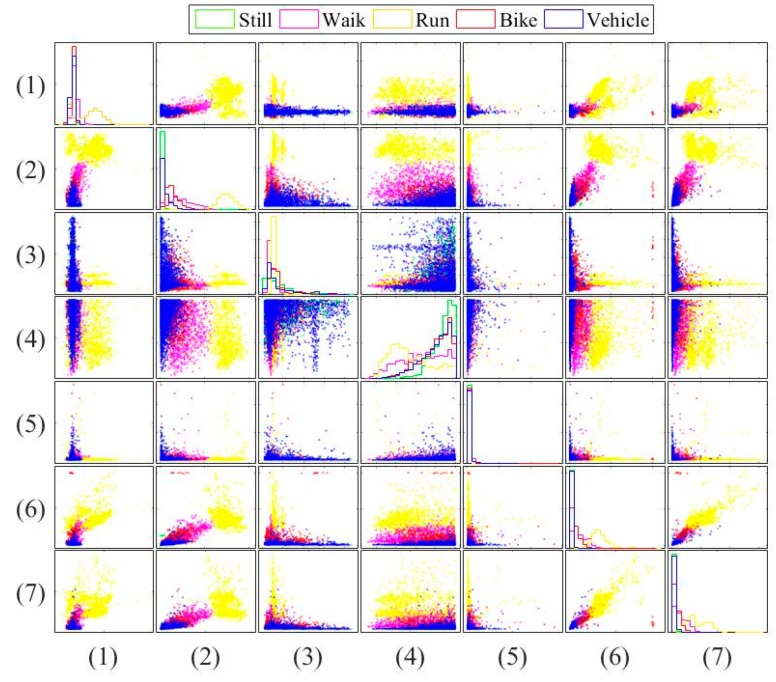
Pairwise comparison of the traditional features in transportation mode.

**Figure 6:**
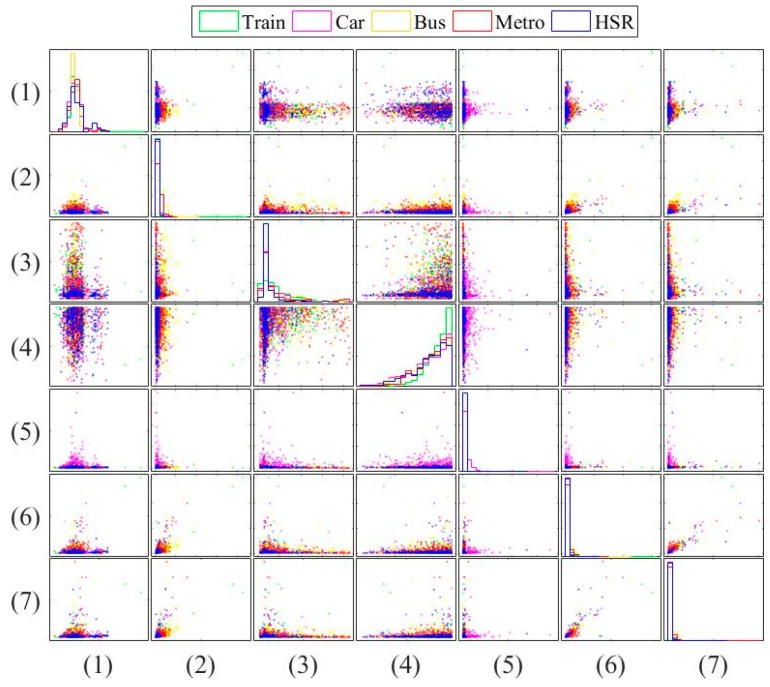
Pairwise comparison of the traditional features in vehicular mode.

Next, Figure 5 and Figure 6 show the pairwise comparison of the traditional features in transportation and vehicular modes, respectively. These figures can identify the ability of each feature. For example, Figure 5 shows that the fourth feature outperforms the fifth one in transportation mode classification. Figure 5 and Figure 6 again show that the vehicular mode classification is more difficult than that in transportation mode. It motivates us to use more features. To improve accuracy for both transportation and vehicular modes, we select and combine useful features from existing works. However, due to the constrained power and resources, the modification and dimension should be minor.

The aim of this study is not to propose a new statistic feature, which has been well investigated. Instead, we try to select and combine useful features from existing works under the power and dimension constrain for both transportation and vehicular modes classification tasks. In fact, we have tried thousands subset combinations heuristically in the experiments of this study, and reported the best one as the proposed feature. Next, this paper fetched six notable features based on the above-mentioned features and Liu et al. [20], and then figured out other eight features. This paper combines these 14 features to classify training and testing data to evaluate the accuracy. Note that among the proposed features, the first four features were proposed by [5], and the fifth and sixth were derived from [20]. The proposed features are described as followed:

(1) Average of the accelerometer’s magnitude.
(2) Standard deviation of the accelerometer’s magnitude.
(3) The highest FFT value of the accelerometer.
(4) Average of the gyroscope’s value.
(5) Acceleration in the z direction compared with gravity.
(6) Horizontal section (X-Z plane) of the accelerometer’s magnitude.
(7) Average of the X direction of acceleration.
(8) Average of the Y direction of acceleration.
(9) Average of the Z direction of acceleration.
(10) Maximum of the accelerometer’s magnitude.
(11) Average of acceleration instantly changes.
(12) Standard deviation of acceleration instantly changes.
(13) Average of the magnetometer’s value.
(14) Average of magnetic instantly changes.

### 2.3. Machine Learning Algorithms

With the training data from Section 2.2, this paper then used three machine learning algorithms, including decision tree, K-nearest neighbor, and support vector machine, to train classifier. The following is the introduction of each algorithm.

#### 2.3.1. Decision Tree (DT)

The DT algorithm exemplifies every possible outcome of a decision through means of categorizing the data in each step for regression and classification. In the DT algorithm, a tree is created by a specific algorithm, which is a supportive tool used to simplify a given set of complex data. The decision tree consists of nodes and branches based on a rule. The nodes illustrate that a decision has been made whilst the branches that spread to the left or right from the nodes show that the data is further being categorized. On each occasion when a decision has been made, a new counter node is formed. This in effect forms the ‘tree-like’ graph to help individuals visually analyze the data so that an accurate and meaningful decision can be derived.

A tree searches through variable to find a value of a variable which splits the data into two or more groups. The best split minimizes the error (impurity) in the resulting subsets. To find the best split, we have to measure the degree of impurity of the child nodes [21]. The higher the impurity, the less skewed the class distribution will be. There are several ways to measure the impurity of the best split. Some of the impurity measures are:

- Entropy:
  (1) $$H\left(x\right)=-\overset{n}{\underset{i=1}{\sum }}p_{i}\;{\text{log}}_{2}\;p_{i}$$
- Gini Impurity: impurity-based metrics which is used to measure how often an element from a set can be labeled incorrectly. It can be measured as:
  (2) $$Gini\;Impurity=1-\underset{i}{\sum }p_{i}^{2}$$
  (3) $$Classification\;Error=1-max(p_{i})$$

In Equations (1)–(3), $p_{i}$ is the probability mass function of the *i*-th sample. Compared to other classification algorithms, decision trees are simple to understand, easy to interpret and robust against skewed distributions but a small change can alter the results drastically. One more problem with the decision tree is that they can overfit easily [22].

#### 2.3.2. K-Nearest Neighbor (KNN)

**Figure 7:**
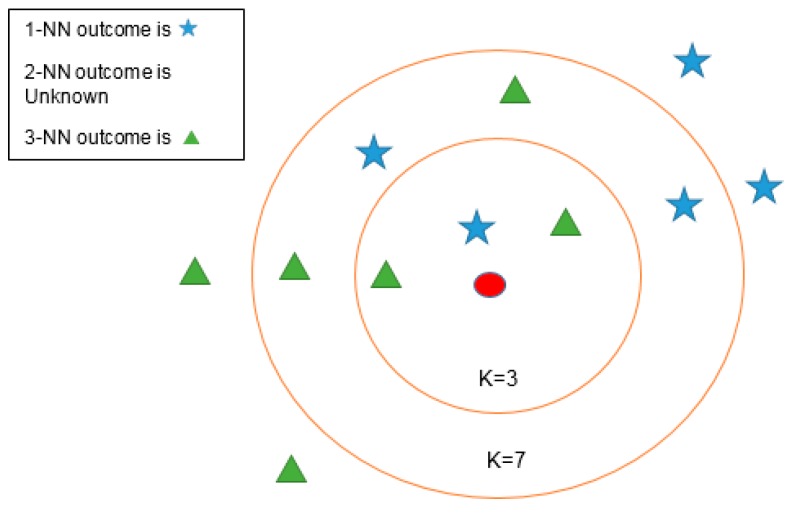
A K-nearest neighbor model.

The KNN algorithm is a non-parametric method used for classification or regression, and the output solely depends on which of the two are being used. For the output in classification an object is usually classified by the majority of the votes received by its neighbors and for the output in regression the object is based on the property value. KNN is a lazy learning algorithm which does not use training data and classifies the new instances based on similarity measure (i.e., distance measure). It classifies the unlabeled instance to the most common node amongst its nearest neighbors based on the distance. Since there is no prior knowledge available in KNN, the decision rule of KNN is dependent on the distance metrics. A simple case of KNN is shown in Figure 7, where a new instance is classified based on the value of K.

The performance is totally dependent upon the way the distances are computed. The distance can be computed using one of the following methods:

(4) $$D\left(x,y\right)=\{\begin{array}{c} \sqrt{\underset{i}{\sum }{\left(x_{i}-y_{i}\right)}^{2}}\\ \underset{i}{\sum }\left|x_{i}-y_{i}\right|\\ {\left[\underset{i}{\sum }{\left(\left|x_{i}-y_{i}\right|\right)}^{p}\right]}^{1/p} \end{array}\;\begin{array}{c} \text{Euclidean Distance}\\ \text{Minkowski Distance}\\ \text{Manhattan Distance} \end{array}$$

where D(x,y) is the shortest distance between any two samples. The most commonly used distance metric is the Euclidean distance. It should also be noted down that the above mentioned three distance metrics are only used for continuous variable. In discrete or categorical case, the Hamming distance is used. Despite it being robust and effective for coping with large training data, the weakness lies in the run time performance with it being considered poor for a large training set and high computational cost.

#### 2.3.3. Support Vector Machine (SVM)

**Figure 8:**
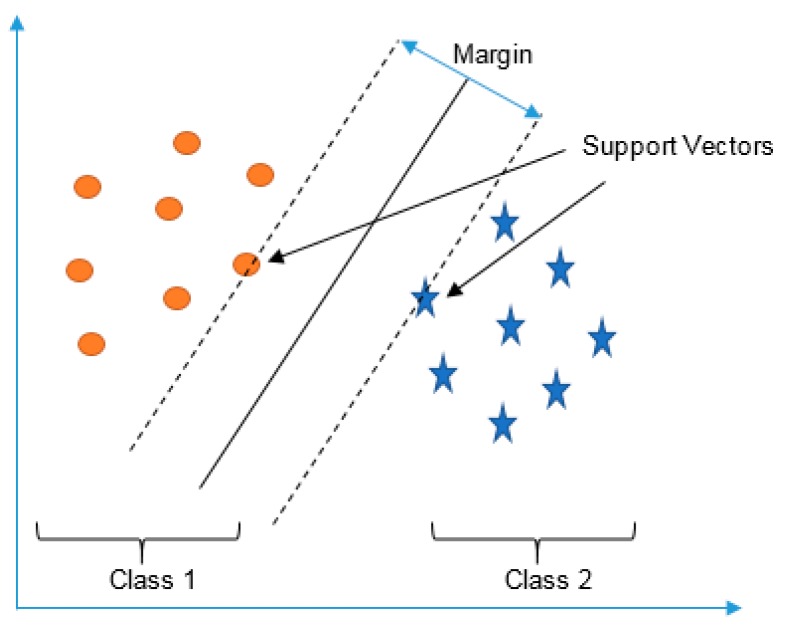
A SVM structure.

SVM is a very popular method, capable of performing classification and regression. It offers very promising results and can capture complex relationships without going into the difficult transformations. SVM constructs a set of hyperplanes in high-dimensional space to separate categories of examples. With these separated categories, people can find obvious differences of each category and classify unknown examples into specific group more accurately. A good separation can be achieved by hyperplanes that has largest functional margin which in return lowers the generalization error. In SVM, a decision surface is to be find which is far from any data point. A simple scenario for support vectors and margin is shown in Figure 8, where the support vectors are the points fall within the margin.

To maximize the margin for a given set of training data, the following optimization problem need to be solved:

(5) $$\underset{w}{\text{min}}\frac{1}{2}{\left\|w\right\|}_{2}^{2}+C\;\overset{N}{\underset{i=1}{\sum }}\epsilon_{i}$$

(6) $$\text{subject to}\;y_{i}\left(w^{T}x_{i}+b\right)\geq 1-\epsilon_{i},\;\forall x_{i}$$

$$\epsilon_{i}\geq 0$$

where $y_{i}$ is either 1 or −1, indicating the class to which the point $x_{i}$ belongs. The parameter w is the (not necessarily normalized) normal vector to the hyperplane. The parameter *C* is the regularization parameter used to prevent overfitting. The parameter b determines the offset of the hyperplane from the origin along the normal vector w.

## 3. Results

### 3.1. Transportation Mode Classification

This paper extracts 90,000 feature vectors of each mode and uses three machine learning algorithms to train classification models. This paper compares the results between two sets of features: one is the seven features based on Reference [5], and the other one is the proposed features. First, this paper created two tables to show the performance of each algorithm. Table 2 shows the general accuracy, prediction time, and model size of each algorithm with seven features based on Reference [5] while Table 3 shows that with the proposed 14 features.

In these tables, the general accuracy means the ratio of the correct results to the total testing numbers, the prediction time means how long it would it take for each prediction with the unit of microseconds (i.e., 10^−6^ s), and the model size means the size of each model with the unit of megabits (MB). The results show that DT reports the lowest prediction time and the smallest model size. On the other hand, SVM provides the best performance in accuracy whereas it incurs the largest prediction time. More importantly, the table shows that the proposed features significantly enhance the accuracy in the three machine learning algorithms. Specifically, DT improves from 74.65% to 79.59%, KNN improves from 77.33% to 86.86%, and SVM improves from 81.60% to 86.94%. While using the proposed features, KNN shows a comparable performance to SVM and a slightly larger model size.

**Figure 9:**
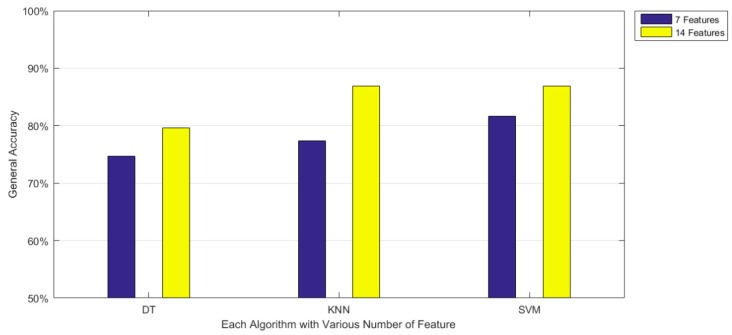
Comparison between two different feature sets on accuracy of three machine learning algorithms.

Next, Figure 3 more clearly compares the two different feature sets on accuracy, showing that when the number of features changes from seven to 14, the accuracy obviously improves. The improvement is the most significant with the KNN method. Based on Figure 9, we can see that SVM has the best performance in general accuracy. Nevertheless, from the other operating points of view, KNN would be also a good choice because of its comparable accuracy and lower prediction time.

For analyzing results more intuitively, this paper constructs the confusion matrices of each algorithm with the proposed 14 features. In these confusion matrices (i.e., Table 4, Table 5 and Table 6), the header columns are the actual label, and the header rows are the prediction label. For instance, if a prediction result is the still mode, and its actual label also is the still label, then this prediction result is correct. If a prediction result is the biking mode, but its actual label should be walking, then people can know that the walking data was misattributed to the bicycle instead. In these confusion matrices, we can find that in many cases, the “in vehicle” data were usually misjudged as “on bike” and “still”. Besides, the running mode always produces the most accurate result. This is because running makes the smartphone shaken severely, making the classification easy.

### 3.2. Vehicle Mode Classification

**Figure 10:**
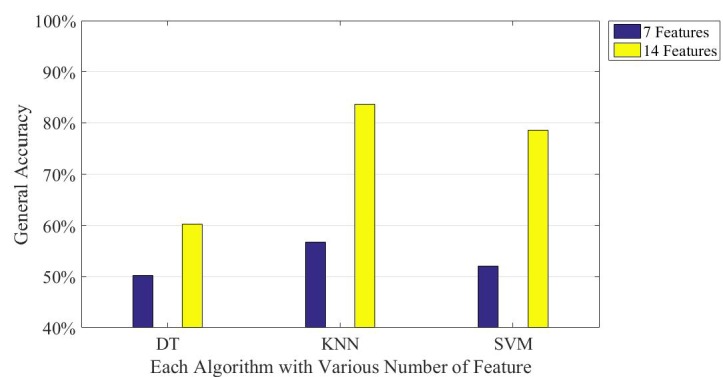
Comparison between two different numbers of features on vehicle mode detection accuracy of three machine learning algorithms.

The vehicle mode includes HSR, metro, bus, car, and train. Table 7 and Table 8 compare the results between the two feature sets; one is the seven features based on Reference [5] (Table 7), and the other one is the proposed features (Table 8). The tables show that the proposed features significantly enhance the accuracy in the three machine learning algorithms. These results from the vehicular mode classification are consistent with that of transportation mode detection. Again, DT reports the lowest prediction time and the smallest model size. The only difference is that KNN provides the best performance in accuracy whereas it also incurs the largest model size. Figure 10 more clearly compares the two different feature sets on accuracy, verifying that when using the proposed features, the accuracy still clearly improves.

**Figure 11:**
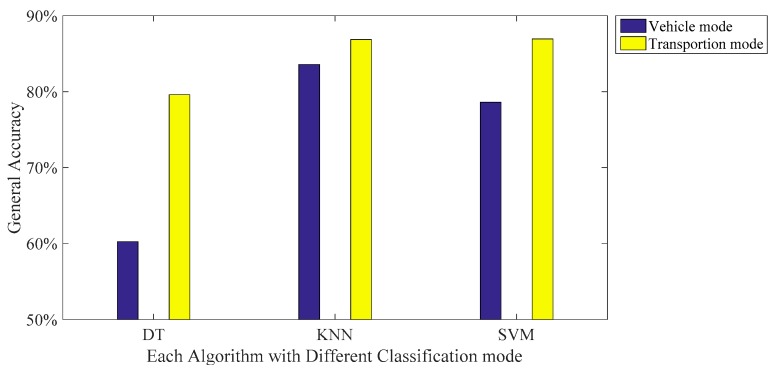
Performance comparison between transportation and vehicle mode classification using three machine learning algorithms.

Similar to the previous transportation mode detection results, Table 9, Table 10 and Table 11 provide the confusion matrices of each algorithm with the proposed features. The results show that among the five vehicular modes, detecting the car mode reports the highest accuracy (89.21% using KNN). On the other hand, the most significant errors occur while classifying the car and train (11.27% using KNN). Figure 11 compares the performance between the transportation and vehicle mode classification. This figure shows that classifying the vehicle mode is more difficult than the transportation mode. The general accuracy reduces from 86.94% to 78.59% and from 86.86% to 83.57%, respectively, based on SVM and KNN. This is because the behaviors of the car-bus and the train-metro are very similar, making the mode classification difficult.

**Figure 12:**
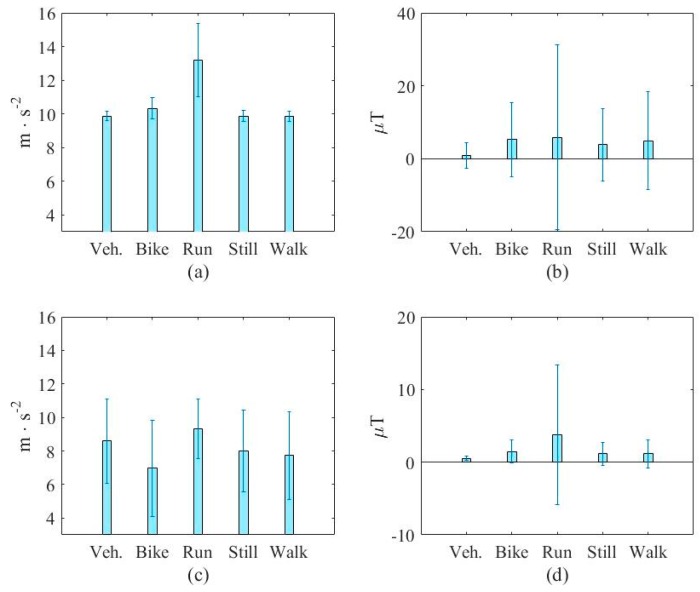
A typical added feature and comparison with an original one in transportation mode. (**a**) The first original feature (average of accelerometer’s magnitude); (**b**) The fifth original feature (standard deviation of magnetometer’s magnitude); (**c**) The sixth added feature (horizontal section (X-Z plane) of the accelerometer’s magnitude); (**d**) The 14th added feature (average of magnetic instant change).

**Figure 13:**
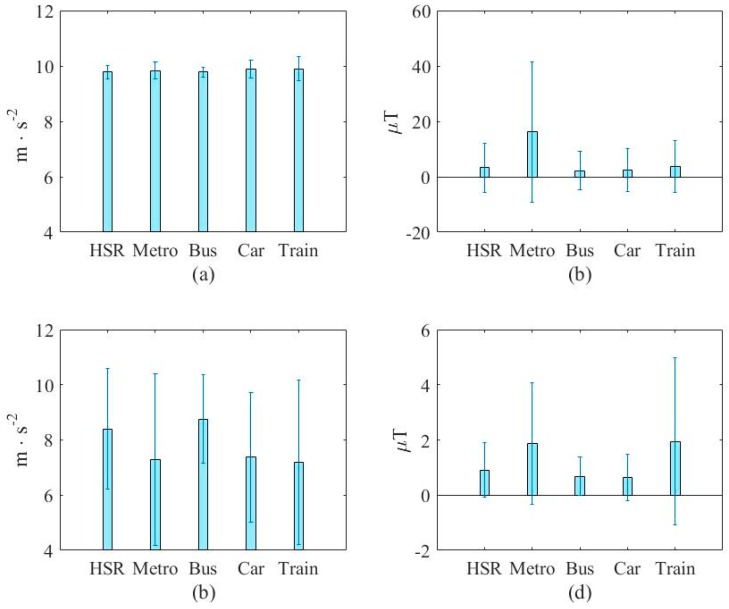
A typical added feature and comparison with an original one in vehicular mode. (**a**) The first original feature (average of accelerometer’s magnitude); (**b**) The fifth original feature (standard deviation of magnetometer’s magnitude); (**c**) The sixth added feature (horizontal section (X-Z plane) of the accelerometer’s magnitude); (**d**) The 14th added feature (average of magnetic instant change).

Figure 7 and Figure 8 show a typical case of the added features and compare them with an original one in the transportation and vehicular modes, respectively. Figure 12a,b show the mean of the first (average of accelerometer’s magnitude) and the fifth original features (standard deviation of magnetometer’s magnitude) from the large database, respectively. For fair comparison, Figure 7c,d show the added features with same unit. Figure 13c,d show the mean of the sixth (horizontal section (X-Z plane) of the accelerometer’s magnitude) and the 14th added features (average of magnetic instant change), respectively. This figure shows that the added feature can provide assistance to the task due to the different properties. More importantly, the added feature can improve the performance of the vehicular mode detection task, as indicated in Figure 8. From this figure, we can see that the first original features are almost the same in the five modes whereas the sixth added feature can separate the data into two categories. These figures again verify the ability of the added features in enhancing the accuracy in both tasks.

## 4. Conclusions

This paper studies the transportation mode using big data from three smartphone sensors based on three machine learning algorithms and two different feature vectors. From the feature perspective, the results show that the proposed features significantly enhance the accuracy in the three machine learning algorithms, as compared to traditional features. From the classifier perspective, SVM has the best performance in the transportation modes’ prediction accuracy whereas it incurs the largest prediction time. While using the proposed features to predict the transportation modes, KNN shows a comparable performance to SVM and a slightly larger model size. This paper also investigates the vehicle mode classification and compares the results with those of the transportation modes. In the vehicle mode detection tasks, KNN outperforms SVM with a shorter prediction time, but contains largest model size. The future work is to study different features and models to overcome the problem of the misattributed results.

## Figures and Tables

**Table 1:** Database description based on Reference [5].

Transportation Mode		Collection Time (h)
	Still	158
	Walking	141
	Running	79
	Biking	98
On a Vehicle	Motorcycle	163
	Car	208
	Bus	75
	Metro	132
	Train	89
	HSR	106

**Table 2:** General accuracy of each algorithm with seven features based on Reference [5].

	Prediction Accuracy	Prediction Time (µs)	Model Size (KB)
DT	74.65%	0.55	8
KNN	77.33%	107	22,145
SVM	81.60%	7120	9858

**Table 3:** General accuracy of each algorithm with the proposed 14 features.

	Prediction Accuracy	Prediction Time (µs)	Model Size (KB)
DT	79.59%	0.69	32.7
KNN	86.86%	4702.48	45,568
SVM	86.94%	9715.80	44,032

**Table 4:** Confusion matrix of decision tree with the proposed features.

DT		Prediction Label					Accuracy of Each Mode
		Still (240,288)	Walk (269,853)	Run (59,722)	Bike (128,010)	Vehicle (1,905,008)	
Actual Label	Still	88.84%	0.89%	0.27%	0.89%	9.11%	88.84%
	Walk	2.19%	77.55%	0.34%	12.36%	7.56%	77.55%
	Run	0.15%	2.70%	96.01%	0.78%	0.35%	96.01%
	Bike	1.12%	12.71%	0.06%	83.60%	2.51%	83.60%
	Vehicle	8.26%	2.89%	0.08%	10.84%	77.93%	77.93%
General Accuracy		79.59%					

**Table 5:** Confusion matrix K-nearest neighbor with the proposed features.

KNN		Prediction Label					Accuracy of Each Mode
		Still (240,288)	Walk (269,853)	Run (59,722)	Bike (128,010)	Vehicle (1,905,008)	
Actual Label	Still	97.00%	0.61%	0.31%	0.48%	1.60%	97.00%
	Walk	1.17%	92.86%	0.20%	2.92%	2.85%	92.86%
	Run	0.04%	1.09%	98.29%	0.43%	0.15%	98.29%
	Bike	0.63%	2.59%	0.15%	94.09%	2.54%	94.09%
	Vehicle	5.39%	3.69%	0.50%	6.54%	83.89%	83.89%
General Accuracy		86.86%					

**Table 6:** Confusion matrix support vector machine with the proposed 14 features.

SVM		Prediction Label					Accuracy of Each Mode
		Still (240,288)	Walk (269,853)	Run (59,722)	Bike (128,010)	Vehicle (1,905,008)	
Actual Label	Still	95.49%	0.50%	0.30%	0.54%	3.16%	95.49%
	Walk	2.24%	86.22%	0.15%	5.30%	6.08%	86.22%
	Run	0.10%	1.40%	97.48%	0.56%	0.46%	97.48%
	Bike	0.90%	2.93%	0.04%	93.67%	2.46%	93.67%
	Vehicle	7.08%	1.77%	0.17%	5.81%	85.18%	85.18%
General Accuracy		86.94%					

**Table 7:** General accuracy of each algorithm with seven features based on Reference [5].

	Prediction Accuracy	Prediction Time (µs)	Model Size (KB)
DT	50.23%	0.81	28
KNN	56.74%	548	25,732
SVM	52.12%	8,362	12,637

**Table 8:** General accuracy of each algorithm with the proposed 14 features.

	Prediction Accuracy	Prediction Time (µs)	Model Size (KB)
DT	60.26%	0.72	40
KNN	83.57%	9,550	106,300
SVM	78.59%	19,550	85,800

**Table 9:** Confusion matrix of the decision tree with the proposed features.

DT		Prediction Label					Accuracy of Each Mode
		HSR (177,963)	Metro (199,030)	Bus (99,343)	Car (484,668)	Train (175,899)	
Actual Label	HSR	56.63%	7.87%	4.83%	14.62%	16.04%	56.63%
	Metro	17.40%	51.41%	2.69%	10.67%	17.84%	51.41%
	Bus	8.85%	3.14%	52.99%	30.47%	4.56%	52.99%
	Car	11.09%	3.27%	11.70%	69.92%	4.03%	69.92%
	Train	19.59%	7.82%	5.46%	15.71%	51.41%	51.41%
General Accuracy		60.26%					

**Table 10:** Confusion matrix for K-nearest neighbor with the proposed features.

KNN		Prediction Label					Accuracy of Each Mode
		HSR (240,288)	Metro (269,853)	Bus (59,722)	Car (128,010)	Train (1,905,008)	
Actual Label	HSR	75.65%	8.73%	2.27%	8.48%	4.92%	75.65%
	Metro	4.21%	86.03%	2.01%	4.06%	3.69%	86.03%
	Bus	3.36%	3.40%	75.69%	11.27%	6.26%	75.69%
	Car	2.96%	2.55%	3.42%	89.21%	1.86%	89.21
	Train	4.95%	5.85%	3.07%	8.42%	77.72%	77.72%
General Accuracy		83.57%					

**Table 11:** Confusion matrix for support vector machine with the proposed features.

SVM		Prediction Label					Accuracy of Each Mode
		HSR (240,288)	Metro (269,853)	Bus (59,722)	Car (128,010)	Train (1,905,008)	
Actual Label	HSR	73.80%	8.36%	2.17%	9.78%	5.91%	73.80%
	Metro	6.96%	79.91%	1.63%	5.46%	6.06%	79.91%
	Bus	4.31%	4.40%	68.21%	18.30%	4.77%	68.21%
	Car	4.67%	2.82%	5.11%	84.42%	2.99%	84.42%
	Train	6.78%	7.01%	2.99%	11.49%	71.73%	71.73%
General Accuracy		78.59%					
